# A Radiative Chemical Process for the Methylene Blue Degradation by Natural Convective Nanofluid Flow over an Upright Cone

**DOI:** 10.1155/2023/5549746

**Published:** 2023-06-30

**Authors:** E. Ragulkumar, J. Vinoth Kumar, N. Abirami, P. Sambath, K. K. Viswanathan

**Affiliations:** ^1^Department of Mathematics, SRM Institute of Science and Technology, Kattankulathur, Chennai-603203, Tamil Nadu, India; ^2^Department of Chemistry, SRM Institute of Science and Technology, Kattankulathur, Chennai-603203, Tamil Nadu, India; ^3^Department of Mathematical Modeling, Faculty of Mathematics, Samarkand State University, University Boulevard 15, Samarkand 140104, Uzbekistan; ^4^Department of Applied Mathematics and Informatics, Termez State University, Termez 190100, Uzbekistan

## Abstract

An upstraight cone with nonisothermal surface velocity, temperature, and concentration was investigated using a numerical solution approach to simulate MHD, MB dye, and various nanofluid flows. Numerical evaluation of the flow field equation was carried out using an excellent finite difference method after it has been converted into a dimensionless form. Different heat transfer occurrences were observed depending on temperature, velocity, and concentration when using several types of nanofluids (TiO, Ag, Cu, and Al_2_O_3_*Z*_3_). The amount of MB dye that was degraded by the synthesized nanofluids under the influence of sunlight irradiation was 81.40 percent as a catalyst (carbon nanodots). The parametric analysis of various features of flow fields has been shown using graphs. It was observed that heat is generated from the cone during the sun light irradiation reaction, heat is transferred to MB dye containing nanofluids, and heat interacts with nanofluids and is involved in the chemical reaction with the assistance of electrons. As MB dye degrades in the absence of catalysts (carbon nanodots), it is only 52 percent effective. MB dye is degraded at 81.40 percent, then becomes stable, and takes 120 minutes to degrade in nanofluids containing MB dye with catalysts (carbon nanodots).

## 1. Introduction

The textile, medicine, dyeing, printing, pharmaceutical, paint, paper, and food industries could all benefit from MB dye. The dye used in textiles is the most common and is considered to be one of the most popular clothing dyes. The adhesive properties of MB allow it to be securely attached to cotton fibers and to fabrics used in textile production. Furthermore, it has the potential to be employed in dye-sensitized solar cells, sensors, microbial fuel cells, capacitors, and other devices. Sambath et al. [1] derived numerical solutions based on Crank–Nicholson methods for the transient radiative MHD heat and mass transfer flow past an upstraight cone when a chemical reaction is occurring. The numerical analysis done by Hanif et al. [2] included a magnetic field and radiative heat flux to account for variable viscosities in water-based hybrid nanofluid flows in a cone with an inverted permeable. The flow of two-dimensional water-based nanofluid containing a nonspherical CdTe nanoparticle solution has been investigated by Hanif et al. [3]. The chemical reaction of nanofluids in different geometries was studied by Thameem Basha et al. [4] based on electrohydromagnetic as well as nonuniform heat absorbs/supplies. Thameem Basha et al. [5] used two different configurations of chemically reacting nanofluids to study how the Lorentz force affects fluid transport. Radiative MHD evaluation of a third-grade viscoelastic non-Newtonian fluid external to an isothermal vertical cone at a low temperature was carried out by Gaffer et al. [6]. Sulochana et al. [7] investigated thermal as well as mass transference across a cone with radiation as well as chemical processes through magnetohydrodynamic streams. Reddy et al. [8] explored heat as well as species transmission properties using an upright cone flow in a porous nanofluid. The work by Sreedevi et al. [9] includes both heat and mass transfer studies of nanoparticles that are constituted of single and multiwall carbon nanotubes near the surface of porous media showing convectional boundary conditions caused by chemical reactions as well as by suction or injection. Hydromagnetic nanofluid flow accompanied by nonuniform heat sources/sinks is described by Basha et al. [10] using two different types of configurations. Using a vertical cone as a testbed, Sambath et al. [11] investigated the laminar free convection of inviscid viscosity electrically conducting fluid. The impact of thermic radiation/chemical processes on thermal and species transport in a flow of nanofluids upon a plumb cone was investigated by Reddy et al. [12]. The plain convective periphery layer heat and mass transport properties of nanofluids around a vertical cone were demonstrated by Sudarsana Reddy and Chamkha [13]. A mathematical model developed by Sandeep and Reddy [14] assessed the heat transfer characteristics of electrically conducting MHD nanofluid flow over a cone and wedge by taking into account nonlinear thermal radiation and heat source/sink effects. The mixed convection flow of a Newtonian fluid through a revolving vertical cone immersed in porous media was carried out by Mallikarjuna et al. [15] while associated thermal and mass transit were also examined. The peripheral layer current of such MHD Eyring–Powell nanofluid was proposed by Babu et al. [16] in terms of buoyancy effects and absorbs/supplies through a permeable cone. As a new carbon precursor, *Indigofera tinctoria* leaf extract was employed by Jothi et al. [17] for the synthesis of nitrogen-functionalized carbon nanodots by a low-cost hydrothermal method. A simple hydrothermal synthetic approach for producing luminous carbon dots was described by Vinoth Kumar et al. [18] utilizing an abiogenous precursor of rice bran without the use of any surface passivating agents. Among many methods to remove organic and inorganic pollutants from industrial effluents, photocatalysis is an extremely well known one. As described by Zhang et al. [19], dyeing has mainly been used in the textiles, paints, and leather industries. Methylene blue (MB) is a colourant that is widely utilised in a variety of sectors. It causes allergic dermatitis, eye burns, mutations, cancer, and skin irritation in animals as well as humans as examined by researchers [20, 21]. Since the medicinal, dyestuffs, and printing industries have expanded fast, raw sewage is being dumped into rivers and lakes on a daily basis, endangering both humans and marine life. Thus, using contemporary technology to treat natural plant dye-containing industrial effluent has become critical, as discussed by Asuntha et al. [22]. As explained by Carneiro et al. [23], there are a number of ways to remove noxious contaminants emitted by waste effluents. The adsorbent capacity of microplastics in sewage sludge may differ for different metal irons and plastic types based on the study conducted by Li et al. [24]. Hao et al. [25] investigated the on-chip catalytic degradation capability of organic dyes using rod-like zero-valent iron (rZVI) formed with a novel polydisc nanoassembly structure. A simple and eco-friendly method for preparing ZnFe_2_O_4_ nanoparticles and reduced graphene oxide (rGO) nanoporous composites was constructed by Baynosa et al. [26] who developed a fast and environmentally sustainable process that uses pure water as the solvent and does not require any further heat processing. The numerical solution technique was used by Ragulkumar et al. [27, 28] to investigate the effects of viscidity dissipative, MHD, between different flows of nanofluid within an upright cone with surface temperature and velocity.

A magnetohydrodynamic (MHD) nanofluid flow towards a vertical sinusoidal wavy surface was reported by Zeeshan et al. [29]. A squeezing flow between rotating circular plates using nanofluid bioconvection was developed by Zeeshan et al. [30]. The time-dependent electrically conductive flow with heat and mass transport that contains gyrotactic microorganisms was theoretically investigated by Majeed et al. [31]. The buildup of Cu and TiO_2_ NPs in the engine oil organizes a nanofluid. The authors [32–36] investigated hybrid nanofluid MHD mixed convection in W- and M-shaped porous systems using an artificial neural network, as well as non-Darcian porosity complex wavy enclosing.

This research collectively highlights the single component photocatalytic systems as using an immersed heated vertical cone for removing MB from industrial wastewater. Different photocatalytic systems were also examined for their effects on the degradation of MB. In this study, guidelines will be provided to develop efficient photocatalytic devices for the removal of MB from wastewater by an upright immersed heated cone. The point of using a heated cone is that by absorbing excess heat from sunlight radiation and concentrating it into the liquid, it enables rapid degradation of MB dye from wastewater due to continuous heat transfer.

## 2. Mathematical Modeling

### 2.1. Formulation of the Problem

**Figure 1:**
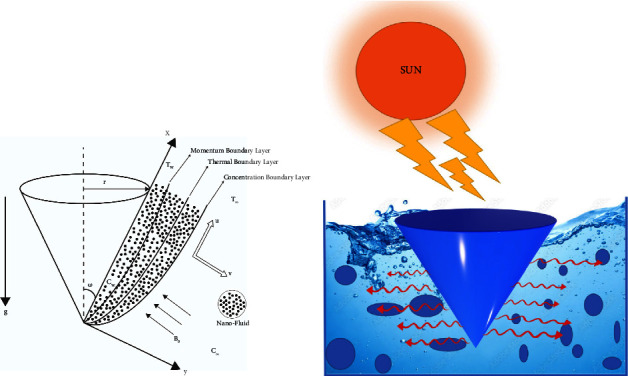
(a) Physical model and (b) cone representation of the overall process.

An investigation of magnetohydrodynamic MB dye with nanofluid flow across a vertical cone was conducted using a nonuniform radiative. A cone's radius and half-angle are determined by *r* and *ω*, respectively.  Figure 1 shows the flow configuration diagram with the *x*-axis equidistant to the cone surface and the *y*-axis ordinary to it. Under the assumption that cone surface thermal reading and species are always lower than *T*_*∞*_ and *C*_*∞*_, Tw > *T*_*∞*_ and Cw > *C*_*∞*_ are calculated. In addition, *T*_*∞*_ and *C*_*∞*_ are the numerical values that represent the ambient temperature and concentration at a distance from the surface, respectively. The third term on the right side of momentum equation (2) consists of a combination of thermal reading and species buoyancy terms, and the final term is the MHD term. A second term on the right side of temperature equation (3) relates to thermal radiation. A first-order chemical reaction is indicated by the end term in diffusion equation (4). The equations for continuity, momentum, energy, and spices, as proposed by researchers [1, 4, 8], may be represented as follows:

Equation of continuity:

(1) $$\begin{array}{c} \frac{\partial \left(ru\right)}{\partial x}+\frac{\partial \left(rv\right)}{\partial y}=0\text{.} \end{array}$$

Equation of momentum:

(2) $$\begin{array}{c} \frac{\partial u}{\partial t^{\prime }}+u\frac{\partial u}{\partial x}+v\frac{\partial u}{\partial y}=g\frac{{\left(\rho \beta_{T}\right)}_{nf}}{\rho_{nf}}\left(T^{\prime }-T_{\infty }^{\prime }\right)\cos\;\omega \\ +g\frac{{\left(\rho \beta_{C}\right)}_{nf}}{\rho_{nf}}\left(C^{\prime }-C_{\infty }^{\prime }\right)\cos\;\omega +v_{nf}\frac{\partial^{2}u}{\partial y^{2}}-\frac{\sigma B_{0}^{2}}{\rho_{nf}}u\text{.} \end{array}$$

Equation of energy:

(3) $$\begin{array}{c} \frac{\partial T^{\prime }}{\partial t^{\prime }}+u\frac{\partial T^{\prime }}{\partial x}+v\frac{\partial T^{\prime }}{\partial y}=\frac{1}{{\left(\varrho c_{p}\right)}_{nf}}\left[k_{nf}\frac{\partial^{2}T^{\prime }}{\partial y^{2}}-\frac{\partial q_{r}}{\partial y}\right]\text{.} \end{array}$$

Equation of concentration:

(4) $$\begin{array}{c} \frac{\partial C^{\prime }}{\partial t^{\prime }}+u\frac{\partial C^{\prime }}{\partial x}+v\frac{\partial C^{\prime }}{\partial y}=D\frac{\partial^{2}C^{\prime }}{\partial y^{2}}-K_{1}\left(C^{\prime }-C_{\infty }^{\prime }\right)\text{.} \end{array}$$

Initial and peripheral conditions are

(5) $$\begin{array}{c} t^{\prime }\leq 0:u=0,v=0,T^{\prime }=T_{\infty }^{\prime },C^{\prime }=C_{\infty }^{\prime }\;for\;all\;x\;and\;y\\ t^{\prime }>0:u=0,v=0,\frac{\partial T^{\prime }}{\partial y}=\frac{-q_{w}x}{k},\frac{\partial C^{\prime }}{\partial y}=\frac{-q_{w}^{*}x}{D}\;at\;y=0\text{,} \end{array}$$

(6) $$\begin{array}{c} u=0,T^{\prime }=T_{\infty }^{\prime }\;C^{\prime }=C_{\infty }^{\prime }\;at\;x=0\\ u=0,T^{\prime }⟶T_{\infty }^{\prime }\;C^{\prime }⟶C_{\infty }^{\prime }\;as\;y⟶\infty \text{,} \end{array}$$

where *q*_*w*_(*x*)=*ax*^*n*^, *q*_*w*_^*∗*^(*x*)=*bx*^*n*^.

The expressions for density *ρ*_*nf*_, heat capacitance (*ρc*_*p*_)_*nf*_, and thermal expansion coefficient (*ρβ*)_*nf*_ for nanofluids are provided by

(7) $$\begin{array}{c} \rho_{nf}=\left(1-\phi \right)\rho_{f}+\phi \rho_{s}\text{,}\\ {\left(\rho \beta \right)}_{nf}=\left(1-\phi \right){\left(\rho \beta \right)}_{nf}=\left(1-\phi \right){\left(\rho \beta \right)}_{f}+\phi {\left(\rho \beta \right)}_{s}\text{,}\\ {\left(\rho c_{p}\right)}_{nf}=\left(1-\phi \right){\left(\rho c_{p}\right)}_{nf}=\left(1-\phi \right){\left(\rho c_{p}\right)}_{f}+\phi {\left(\rho c_{p}\right)}_{s}\text{,} \end{array}$$

(8) $$\begin{array}{c} \frac{k_{nf}}{k_{f}}=\frac{k_{s}+\left(n-1\right)k_{f}-\left(n-1\right)\phi \left(k_{f}-k_{s}\right)}{k_{s}+\left(n-1\right)k_{f}+\phi \left(k_{f}-k_{s}\right)}\text{,}\\ \frac{\mu_{nf}}{\mu_{f}}=\frac{1}{{\left(1-\phi \right)}^{2.5}}\text{.} \end{array}$$

The following nondimensional quantities are discussed:

(9) $$\begin{array}{c} X=\frac{x}{L},Y=\frac{y}{L},Gr^{1/5},U=\frac{uL}{\nu_{f}}Gr_{L}^{-2/5},V=\frac{vL}{\nu_{f}}Gr_{L}^{-1/5}\text{,}\\ t=\frac{\nu_{f}t^{\prime }{\left(Gr_{L}\right)}^{2/5}}{L^{2}},T=\frac{T^{\prime }-T_{\infty }^{\prime }}{\left(q_{w}\left(L\right)L/k\right)}Gr_{L}^{1/5},C=\frac{C^{\prime }-C_{\infty }^{\prime }}{\left(q_{w}\left(L\right)L/D\right)}Gr_{L}^{1/5}\text{,}\\ Gr_{L}=\frac{{\left(g\beta_{T}\right)}_{f}q_{w}\left(L\right)L^{4}\;\cos\;\omega }{k_{f}\;\nu_{f}^{2}},Gr_{C}=\frac{{\left(g\beta_{c}^{*}\right)}_{f}q_{w}^{*}\left(L\right)L^{4}\;\cos\;\omega }{\nu_{f}^{2}\;D_{f}}\text{,}\\ \lambda =\frac{K_{1}L^{2}}{\nu_{f}}Gr_{L}^{-2/5},\mathrm{Pr}\;=\frac{\nu_{f}}{\alpha_{f}},M=\frac{\sigma B_{0}^{2}L^{2}}{\mu_{f}}Gr_{L}^{-2/5},Sc=\frac{\nu_{f}}{D}\text{,}\\ R_{d}=\frac{k_{1}^{*}k}{4\sigma^{*}T_{\infty }^{\prime 3}},R=\frac{r}{L};r=x\;\sin\;\omega \text{.} \end{array}$$

As a result of the Rosseland approximation [11], the radiative flux qr is given by the following expression:

(10) $$\begin{array}{c} q_{r}=-\frac{4\sigma^{*}}{3K^{*}}\frac{\partial T^{4}}{\partial y}\text{.} \end{array}$$

If the term T′-T_*∞*_′ inside the flow is sufficiently small, the linear version of equation (11) can be derived by ascending T^′4^ by utilizing the Taylor series expansion around T_*∞*_′ while removing upper-order terms.

(11) $$\begin{array}{c} T^{4}≅4T_{\infty }^{3}-3T_{\infty }^{4}\text{,} \end{array}$$

(12) $$\begin{array}{c} q_{r}=-\frac{16\sigma^{*}T_{\infty }^{3}}{3K^{*}}\frac{\partial^{2}T}{\partial y^{2}}\text{.} \end{array}$$

The governing equations [27] are given as follows in nondimensional form.

Equation of continuity:

(13) $$\begin{array}{c} \frac{\partial U}{\partial X}+\frac{\partial V}{\partial Y}+\frac{U}{X}=0\text{.} \end{array}$$

Equation of momentum:

(14) $$\begin{array}{c} \frac{\partial U}{\partial t}+U\frac{\partial U}{\partial X}+V\frac{\partial U}{\partial Y}=A_{1}\left[A_{2}\left(T\right)+A_{3}\left(NC-MU\right)\right]+A_{4}\frac{\partial^{2}U}{\partial Y^{2}}\text{.} \end{array}$$

Equation of energy:

(15) $$\begin{array}{c} \frac{\partial T}{\partial t}+U\frac{\partial T}{\partial X}+V\frac{\partial T}{\partial Y}=A_{5}\left[\frac{k_{nf}}{k_{f}}\frac{1}{\mathrm{Pr}}\frac{\partial^{2}T}{\partial Y^{2}}-\frac{1}{\mathrm{Pr}}\left(\frac{3R_{d}+4}{3R_{d}}\right)T\right]\text{.} \end{array}$$

Equation of concentration:

(16) $$\begin{array}{c} \frac{\partial C}{\partial t}+U\frac{\partial C}{\partial X}+V\frac{\partial C}{\partial Y}=\frac{1}{Sc}\frac{\partial^{2}C}{\partial Y^{2}}-\lambda C\text{,} \end{array}$$

where *A*_1_=(1/(1 − *ϕ*)+*ϕ*(*ρ*_*s*_/*ρ*_*f*_)),

(17) $$\begin{array}{c} A_{2}=\left(\left(1-\phi \right)+\phi \frac{{\left(\rho \beta \right)}_{s}}{{\left(\rho \beta \right)}_{f}}\right)\text{,}\\ A_{3}=\left(1-\phi \right)+\phi \frac{{\left(\rho \beta^{*}\right)}_{s}}{{\left(\rho \beta^{*}\right)}_{f}}\text{,}\\ A_{4}=\frac{1}{{\left(1-\phi \right)}^{2.5}\left(1-\phi +\left(\left(\rho_{s}\right)/\left(\rho_{f}\right)\right)\right)}\text{,}\\ A_{5}=\frac{1}{\left(1-\phi \right)+\phi \left({\left(\rho c_{p}\right)}_{s}/{\left(\rho c_{p}\right)}_{f}\right)}\text{.} \end{array}$$

Initial and boundary conditions [28] in nondimensional form are

(18) $$\begin{array}{c} \begin{array}{ccc} t^{\prime }\leq 0:U=0\text{,} & V=0\text{,} & T=0\;for\;all\;X\;and\;Y\text{,}\\ t^{\prime }>0:U=0\text{,} & V=0\text{,} & \frac{\partial T}{\partial Y}=-X^{n},\frac{\partial C}{\partial Y}=-X^{m}\;at\;Y=0\text{,}\\ U=0\text{,} & T=0\text{,} & C=0\;at\;X=0\text{,}\\ U⟶0 & T⟶0 & C⟶0\;as\;Y⟶\infty \text{.} \end{array} \end{array}$$

### 2.2. Engineering Curiosity

Mathematically, the nondimensional coefficient of local skin friction (*τ*_*X*_), the local Nusselt number (*Nu*_*X*_), and the local Sherwood number *Sh*_*X*_ [1] are defined as

(19) $$\begin{array}{c} \tau_{X}=\frac{1}{{\left(1-\phi \right)}^{2.5}}Gr^{3/5}{\left(\frac{\partial U}{\partial Y}\right)}_{Y=0}\text{,}\\ Nu_{X}=\frac{k_{nf}}{k_{f}}\frac{-X{\left(\partial T/\partial Y\right)}_{Y=0}Gr^{1/5}}{T_{Y=0}}\text{,}\\ Sh_{X}=\frac{-X{\left(\partial C/\partial Y\right)}_{Y=0}Gr^{1/5}}{C_{Y=0}}\text{.} \end{array}$$

## 3. Method of Numeric Solution

Crank–Nicolson form FDEs are used with initial and boundary conditions (18) to solve the unstable, nonlinear PDEs (13)–(16). A dimensionless equation was converted into a finite difference equation by using the appropriate finite difference operator with grid discretization. The finite difference equations for velocity, temperature, and concentration are produced by utilizing specifically chosen constant values. To convert finite difference equations into algebraic equations, we need to obtain them. These algebraic equations are presented as a tridiagonal system, and their solutions are found using the Thomas technique. The size of each step in both directions ∆*X* and ∆*Y* is 0.05, with a time step of *t* = 0.01, and X_max_=1 and Y_max_=20 demonstrate the boundary conditions associated with *y*⟶*∞*. First, the spatial mesh sizes are reduced by 50% in one direction and then by 50% in both directions to execute the calculations. Comparing and contrasting the results is done. It is crucial for one to understand that in every case, the results only differ at the fifth decimal place. As a result, the decision of mesh size appears to be appropriate. The *U*_*i*,*j*_^*k*^ and *V*_*i*,*j*_^*k*^ coefficients that appear in the finite-difference equations are treated as constants at each time step. The grid points *i*, *j*, and *k* are all situated along the *X*, *Y*, and time axes, respectively. The values of *U*, *V*, and *T* are known at any grid point when *t* = 0 from the initial conditions with grid discretization, which is given by

(20) $$\begin{array}{c} \frac{U_{i,j}^{k+1}-U_{i-1,j}^{k+1}+U_{i,j}^{k}-U_{i-1,j}^{k}+U_{i,j-1}^{k+1}-U_{i-1,j-1}^{k+1}+U_{i,j-1}^{k}-U_{i-1,j-1}^{k}}{4∆X}\\ +\frac{V_{i,j}^{k+1}-U_{i,j-1}^{k+1}+U_{i,j}^{k}-U_{i,j-1}^{k}}{2∆Y}+\frac{U_{i,j}^{k+1}-U_{i,j-1}^{k+1}+U_{i,j}^{k}-U_{i,j-1}^{k}}{4i∆X}=0\text{,} \end{array}$$

(21) $$\begin{array}{c} \frac{U_{i,j}^{k+1}-U_{i,j}^{k}}{∆t}+U_{i,j}^{k}\frac{U_{i,j}^{k+1}-U_{i-1,j}^{k+1}+U_{i,j}^{k}-U_{i-1,j}^{k}}{2∆X}\\ +V_{i,j}^{k}\frac{U_{i,j+1}^{k+1}-U_{i,j-1}^{k+1}+U_{i,j+1}^{k}-U_{i,j-1}^{k}}{4∆Y}\\ =A_{1}\left[\frac{A_{2}}{2}\left(T_{i,j}^{k+1}+T_{i,j}^{k}\right)+\frac{A_{3}}{2}\left(N\left(C_{i,j}^{k+1}+C_{i,j}^{k}\right)-M\left(U_{i,j}^{k+1}+U_{i,j}^{k}\right)\right)\right]\\ +A_{4}\frac{U_{i,j-1}^{k+1}-2U_{i,j}^{k+1}+U_{i,j+1}^{k+1}+U_{i,j-1}^{k}-2U_{i,j}^{k}+U_{i,j+1}^{k}}{2{\left(∆Y\right)}^{2}}\text{,}\\ +V_{i,j}^{k}\frac{T_{i,j+1}^{k+1}-T_{i,j-1}^{k+1}+T_{i,j+1}^{k}-T_{i,j-1}^{k}}{4∆Y}\\ =A_{5}\left[\frac{k_{nf}}{k_{f}}\frac{1}{\mathrm{Pr}}\left(\frac{T_{i,j-1}^{k+1}-2T_{i,j}^{k+1}+T_{i,j+1}^{k+1}+T_{i,j-1}^{k}-2T_{i,j}^{k}+T_{i,j+1}^{k}}{2{\left(∆Y\right)}^{2}}\right)\right.\\ -\left.\frac{1}{\mathrm{Pr}}\left(\frac{3R_{d}+4}{3R_{d}}\right)\left(T_{i,j}^{k+1}+T_{i,j}^{k}\right)\right]\text{,}\\ +V_{i,j}^{k}\frac{C_{i,j+1}^{k+1}-C_{i,j-1}^{k+1}+C_{i,j+1}^{k}-C_{i,j-1}^{k}}{4∆Y}\\ =\frac{1}{Sc}\frac{C_{i,j-1}^{k+1}-2C_{i,j}^{k+1}+C_{i,j+1}^{k+1}+C_{i,j-1}^{k}-2C_{i,j}^{k}+C_{i,j+1}^{k}}{2{\left(∆Y\right)}^{2}}-\frac{\lambda }{2}\left(C_{i,j}^{k+1}+C_{i,j}^{k}\right)\text{.} \end{array}$$

(22) $$\begin{array}{c} \frac{T_{i,j}^{k+1}-T_{i,j}^{k}}{∆t}+U_{i,j}^{k}\frac{T_{i,j}^{k+1}-T_{i-1,j}^{k+1}+T_{i,j}^{k}-T_{i-1,j}^{k}}{2∆X}\\ +V_{i,j}^{k}\frac{T_{i,j+1}^{k+1}-T_{i,j-1}^{k+1}+T_{i,j+1}^{k}-T_{i,j-1}^{k}}{4∆Y}\\ =A_{5}\left[\frac{k_{nf}}{k_{f}}\frac{1}{\mathrm{Pr}}\left(\frac{T_{i,j-1}^{k+1}-2T_{i,j}^{k+1}+T_{i,j+1}^{k+1}+T_{i,j-1}^{k}-2T_{i,j}^{k}+T_{i,j+1}^{k}}{2{\left(∆Y\right)}^{2}}\right)\right.\\ -\left.\frac{1}{\mathrm{Pr}}\left(\frac{3R_{d}+4}{3R_{d}}\right)\left(T_{i,j}^{k+1}+T_{i,j}^{k}\right)\right]\text{,}\\ +V_{i,j}^{k}\frac{C_{i,j+1}^{k+1}-C_{i,j-1}^{k+1}+C_{i,j+1}^{k}-C_{i,j-1}^{k}}{4∆Y}\\ =\frac{1}{Sc}\frac{C_{i,j-1}^{k+1}-2C_{i,j}^{k+1}+C_{i,j+1}^{k+1}+C_{i,j-1}^{k}-2C_{i,j}^{k}+C_{i,j+1}^{k}}{2{\left(∆Y\right)}^{2}}-\frac{\lambda }{2}\left(C_{i,j}^{k+1}+C_{i,j}^{k}\right)\text{.} \end{array}$$

(23) $$\begin{array}{c} \frac{C_{i,j}^{k+1}-C_{i,j}^{k}}{∆t}+U_{i,j}^{k}\frac{C_{i,j}^{k+1}-C_{i-1,j}^{k+1}+C_{i,j}^{k}-C_{i-1,j}^{k}}{2∆X}\\ +V_{i,j}^{k}\frac{C_{i,j+1}^{k+1}-C_{i,j-1}^{k+1}+C_{i,j+1}^{k}-C_{i,j-1}^{k}}{4∆Y}\\ =\frac{1}{Sc}\frac{C_{i,j-1}^{k+1}-2C_{i,j}^{k+1}+C_{i,j+1}^{k+1}+C_{i,j-1}^{k}-2C_{i,j}^{k}+C_{i,j+1}^{k}}{2{\left(∆Y\right)}^{2}}-\frac{\lambda }{2}\left(C_{i,j}^{k+1}+C_{i,j}^{k}\right)\text{.} \end{array}$$

## 4. Results and Discussion

**Figure 2:**
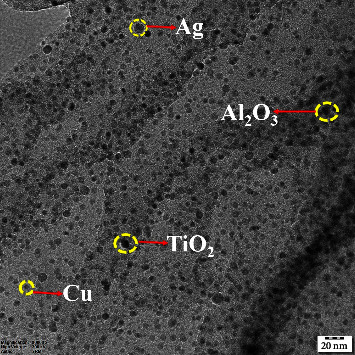
Transmission electron microscopy of nanofluids [17].

**Figure 3:**
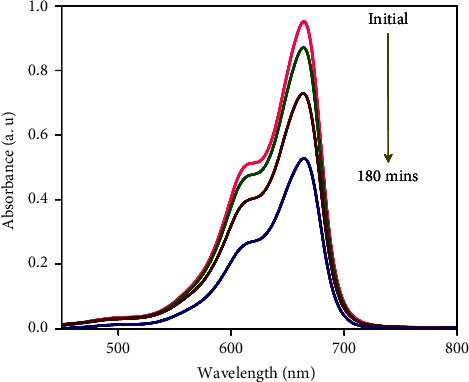
UV-visible spectra of the degradation of MB-using nanofluids in the absence of a catalyst (carbon nanodots).

**Figure 4:**
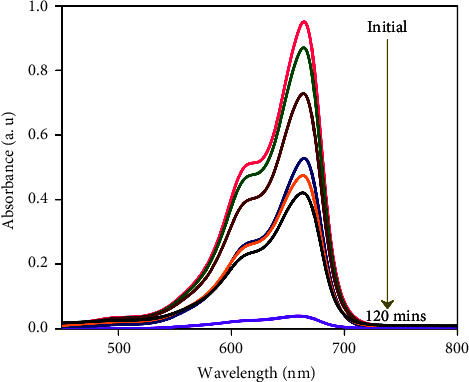
UV-visible spectra of the degradation of MB-using nanofluids in the presence of carbon nanodots.

**Figure 5:**
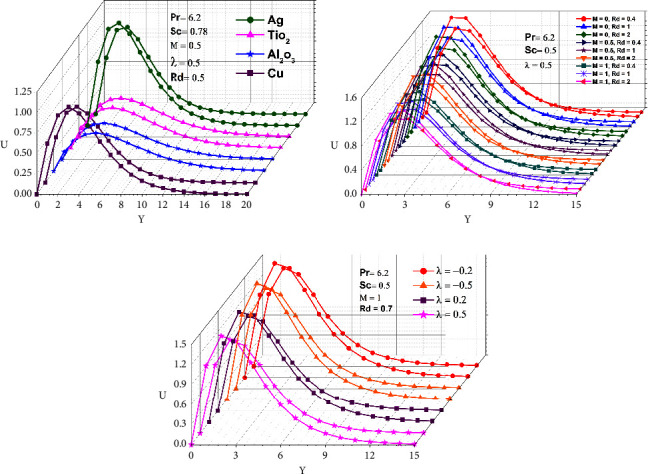
Velocity dispersion of various nanofluids, MHD, radiation, and chemical reactions.

**Figure 6:**
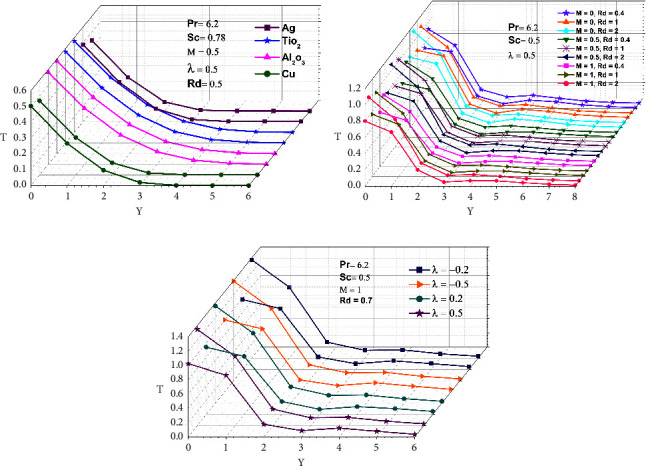
Temperature dispersion of various nanofluids, MHD, radiation, and chemical reactions.

**Figure 7:**
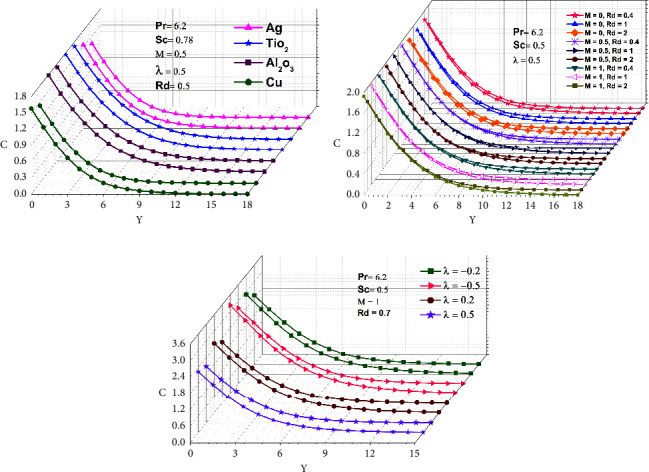
Concentration of various nanofluids, MHD, radiation, and chemical reactions.

In this work, four distinct kinds of nanoparticles, Cu, Ag, Al_2_O_3_, and TiO_2_, are tested as shown in Table 1, employing water as the base nanofluid. As shown in Figures 2–7, transmission electron microscopy of nanofluids, MB-dye degradation, as well as spatial velocity and temperature inside the boundary layer are affected by the magnetic parameter (M), radiation parameter (Rd), chemical reaction (*λ*), and nanoparticles type.  Figure 2 shows the spherical nature of the different nanofluids, and it is confirmed by HR-TEM analysis. In addition, all the nanoparticles are below 20 nm.  Figure 3 shows the catalytic degradation of MB dyes by the formed nanofluids to determine their photocatalytic activity. The UV spectrum confirms the presence of an absorption peak at 664 nm for MB dye. The degradation of MB dye was achieved by photocatalytic intensities induced by different levels of sunlight irradiation without a catalyst (carbon nanodots) and the use of a vertical cone. Furthermore, the MB dye degrades in around 180 minutes in the absence of a catalyst (carbon nanodots) but only to a 52 percent extent. As shown in Figure 4, MB dye-containing nanofluids with a catalyst (carbon nanodots) were immersed in the prepared heated vertical cone, resulting in a significant degradation of the dye intensity.

The heated upright cone is immersed in the liquid containing the nanoparticle-mixed MB dye, and heat transmission to the fluid occurs through the heated cone by irradiating it with sunlight at various time periods. As a result, the heat emitted by the heated cone is absorbed by the nanoparticle, raising the temperature of the liquid and further degrading the MB dye. Finally, the degradation of MB dye happens at 81.40 percent as portrayed in Table 2, after which it becomes stable and takes around 120 minutes at high photocatalytic activity. A comparison of various water-based nanofluid velocities is presented in Figure 5(a). It has been confirmed that a reduction in velocity occurred by using different nanofluids. In contrast to Al_2_O_3_–water, the velocity of the Ag–water nanofluid was higher. Additionally, it can be observed that the magnitude of the buoyancy force is overrun considerably by the mainstream velocity. Similarly, as shown in Figures 6(a) and 7(a), utilizing different nanofluids resulted in a reduction of temperature and concentration. As compared to Cu–water, Al_2_O_3_–water nanofluids exhibited a higher temperature and concentration. This implies that because copper has a high thermal conductivity, heat can move through it fast and the aluminum-water interaction boosts the reaction rate and amount of hydrogen generated. The reaction is slowed down by a larger *λ*, reducing the probability of a successful collision between the reactants.

In Table 3, the coefficient of skin friction and local Nusselt number values for the Prandtl number are compared. Rising (Pr) values signify a decline in the local friction coefficient with growing local Nusselt numbers.

As shown in Figures 5(b), 6(b), and 7(b), M and Rd have a significant effect on the flow, velocity, temperature, and concentration profiles for nine different values. *M* = 0 for lack of magnetic field occurs for no applied magnetic field. All velocities accelerated as M increased (Figure 5(b)). In a boundary layer region, it has been demonstrated that the magnetic force allows for a decreasing boundary layer as the velocity terms remain positive in the momentum equation. The Lorentz force, which is associated with the boundary layer of a magnetic field, gets thinner with an increase in M and Rd. As the radiation and magnetic lines move past the vertical cone, the free stream velocity is regarded as determining how the lines move. As a result of the magnetic field, the fluid that had been decelerated by viscous force is accelerated, which also counteracts the effects of viscous force. Thus, as M and Rd increase, the fluid's velocity decreases as well. Similarly, as demonstrated in Figures 6(b) and 7(b), increasing M and Rd causes a rise in temperature and concentration. Furthermore, for permeable cones, the thermal and species boundary layer thickness rises as M and Rd increase, while temperature and concentration remained the same. The magnetic force is perpendicular to the momentum force, resulting in a uniform circular motion, which causes momentum to decline and the MHD effect to rise. In the case of the increasing wall temperature rate, the MHD and thermal radiation effects can be incorporated. As a result, a voltage is induced if a conductor is moved relative to a magnetic field, resulting in a current flowing between the terminals, which emits high-frequency electromagnetic radiation in comparison to low-frequency radiation. Chemical reactions that result in an increase due to the average kinetic energy of molecules increase the thickness of the thermal boundary layer.

Figures 5(c), 6(c), and 7(c) demonstrate that when comparing the three factors, velocity, temperature, and concentration all start rising when the value of lambda (*λ*) begins to fall because more molecules or ions interact to form new compounds and the rate of reaction increases. A decrease in species boundary peak thickness is evident as M and Rd values increase. The reason behind this is that the involvement of a field of magnetism in a liquid that conducts electricity provides a force known as the Lorentz force, which opposes the flow of direction as well as causes depreciation in concentration profiles. Hence, chemical reactions have a tendency to lower mass transfer rates at the surface cone. This happens because more molecules or ions interact when the concentration of all reactants increases, resulting in a faster reaction rate.

## 5. Conclusion

In this work, we used numerical simulations to examine the process of radiative chemical reaction for the degradation of methylene blue using free convective nanofluid flow past an upright cone. The illustrations indicate that there are fluctuations in velocity, temperature, and concentration based on the absorbance of the nanoparticles, which occurs most commonly during heat and mass transfer. As a result, Al_2_O_3_ in Figures 6(a) and 7(a), whereas Ag in Figure 5(a), has a significantly larger temperature, concentration, and velocity. The given Figures (5(b), 6(b), 7(b), 5(c), 6(c), and 7(c)) show that the magnetic field, radiation, and chemical reaction dominate the liquids and their values rise. The nanoparticles present may produce overheat, thus increasing their velocity, temperature, as well as concentration, and their effect can be a precursor to the decay of the MB dye. The effectiveness of nanomaterials as catalysts from an environmental perspective analyzed for the degradation of MB by using a catalyst and heated upright cone for water degradation was attained within 120 min. Overall reports suggest that nanofluids containing MB dye can be converted to 81.40% water degradation and used for environmental remediation applications. In addition, our future research direction is towards free convection nanofluid flow past a vertical wavy cone mediator for methylene blue degradation with MHD radiative chemical reaction and mass transfer.

## Figures and Tables

**Table 1:** Water-based nanoparticles properties [27] for thermophysical.

Physical features	Water	Copper	Silver	Aluminum oxide	Titanium dioxide
$\bar{\rho }\left(kg/m^{3}\right)$	997.10	8933.0	10500	3970.0	4250.0
${\bar{C}}_{\bar{p}}\left(J/kgK\right)$	4179.0	385.0	235	765.0	686.2
$\bar{k}\left(W/mk\right)$	0.613	401.0	429	40.0	8.9538
$\bar{\beta }\times 10^{-5}\left(K^{-1}\right)$	21.0	1.67	1.89	0.85	0.90

**Table 2:** Table of comparison for the degradation of dyes.

Catalysts	Dyes	Removal efficiency (%)	References
Hierarchical Cu_2_O	MB	55	[37]
Cu_2_O-rGO	MB	72	[38]
CuO-nanozeolite X	MB	68	[39]
Eu3^+^ doped In_2_O_3_	MB	56	[40]
Carbon nanodots	MB	81.40	Present result

**Table 3:** Local skin friction and Nusselt number are compared with those of Hossain and Paul [41] as well as Ragulkumar et al. [28] for different Pr values.

Pr	Local skin friction			Local Nusselt number		
	Hossain and Paul [41]	Ragulkumar et al. [28]	Present result	Hossain and Paul [41]	Ragulkumar et al. [28]	Present result
0.01	5.13457	5.15625	5.15628	0.14633	0.14594	0.14597
0.05	2.93993	2.95712	2.95721	0.26212	0.26155	0.26158
0.1	2.29051	2.30639	2.30641	0.33174	0.33090	0.33094

## Data Availability

The data used to support the findings of this study are included within the article.

## Nomenclature

*B*_0_: Magnetic field induction (W·m^−2^)
*C*_*p*_: Specific heat at constant pressure (J·kg^−1^·K^−1^)
*C*′: The concentration in the fluid
*C*_*∞*_′: The concentration far away from cone surface
*D*: The thermal diffusivity (m^−2^·s^−1^)
Gr_*L*_: Thermal Grash of number (−)
*g*: Acceleration due to gravity (m·s^−1^)
*k*: Thermal conductivity (W·K^−1^·m^−1^)
*K*_1_: Chemical reaction parameter (dimensional)
*L*: Reference length (m)
Nu_*x*_: Nondimensional local Nusselt number
Pr: Prandtl number (−)
qr: Thermal radiation (dimensional)
*R*: Dimensionless local radius
*R*_*d*_: Radiation (−)
Sc: Schmidt number (−)
Sh_*x*_: Nondimensional local Sherwood number
*T*′: Temperature (k)
*T*: Dimensionless temperature
*t*′: Time (s)
*t*: Dimensionless time
*U*, *V*: Dimensionless velocity in *X*, *Y* direction
*u*, *v*: Dimensional velocity in the direction of *x*, *y* axes (ms^−1^)
*x*: Spatial coordinate alongside the cone generator (dimensional), (m)
*y*: Spatial coordinate perpendicular to cone generator (dimensional), (m)
*X*: Spatial coordinate alongside the cone generator (nondimensional)
*Y*: Spatial coordinate perpendicular to cone generator (nondimensional)

### Greek Symbols

*β*: Volumetric thermal expansion coefficient with temperature (K^−1^)
*λ*: Chemical reaction parameter (−)
*ϕ*: Nanoparticles volume fraction (−)
*μ*: Dynamic viscosity (kg·m^−1^·s^−1^)
*μ*_*nf*_: Nanofluid of the dynamic viscosity (kg·m^−1^·s^−1^)
*ν*: Kinematic viscosity (m^−1^·s^−1^)
*ν*_*f*_: Base fluid of the kinematic viscosity (m^−1^·s^−1^)
*ωω*: Cone apex half-angle (degree)
*ρ*: Density (kg·m^−3^)
*τ*_*X*_: Dimensionless local skin friction

### Subscripts

*f*: Base fluid condition
nf: Nanofluid condition
*s*: Nanoparticles condition
*w*: Wall condition
*∞*: Ambient condition.
